# Polymorphonuclear Cell Functional Impairment in Relapsing Remitting Multiple Sclerosis Patients: Preliminary Data

**DOI:** 10.1371/journal.pone.0131557

**Published:** 2015-06-29

**Authors:** Valeria Allizond, Sara Scutera, Silvia Rossi, Tiziana Musso, Cristina Crocillà, Paola Cavalla, Claudia Trebini, Elisa Simona Marra, Anna Maria Cuffini, Giuliana Banche

**Affiliations:** 1 Bacteriology and Mycology Laboratory, Department of Public Health and Pediatrics, University of Torino, Turin, Italy; 2 Immunology Laboratory, Department of Public Health and Pediatrics, University of Torino, Turin, Italy; 3 ASL TO3 Rivoli Hospital, Rivoli (Turin), Italy; 4 Department of Neuroscience, University of Torino, Turin, Italy; Universitá Cattolica del S. Cuore, ITALY

## Abstract

Multiple Sclerosis patients run an increased risk of microbial infections, which leads to high rates of hospitalization and infection-related mortality. Although immunotherapy may increase infection risk in some cases, data as to the relationship among microbial factors, immunotherapy and alterations in the innate immunity of these patients are still scanty. On these grounds, this interdisciplinary study aims at investigating the role the functional activity of polymorphonuclear cells (PMNs) play in relapsing remitting multiple sclerosis at different stages. The *in vitro* ability of PMNs from patients, either untreated or treated with immunosuppressant or immunomodulatory drugs to kill *Klebsiella pneumonia* or *Candida albicans*, were investigated and compared to PMNs from healthy subjects. The release of various cytokines was also assessed, as was the production of reactive oxygen species and their ability to regulate apoptosis after microbial stimulation. Our results indicate that although patients have a normal number of PMNs, they have a statistically significant (p<0.05) reduction in intracellular killing activity. Although variations are strongly related to the therapeutic management of patients, they are independent from their disease stage. As no statistically significant differences were observed between patients and controls in cytokine release values, reactive oxygen species production or apoptosis, we came to the conclusion that other factors may be involved. Supportive validation of these results from further studies might well help in identifying a subset of patients at high risk of infection who could benefit from a closer follow-up and/or antibiotic prophylaxis.

## Introduction

A growing body of evidence indicates that the central nervous system (CNS) and the immune system are two closely linked super-systems whose functional interaction could explain the appearance of immunological manifestations due to CNS injury and vice versa. Likewise, the emergence of systemic infections after acute brain damage could be a symptom of a CNS-mediated decrease in immune competence [1]. Therefore, patients with CNS injury are at high risk of developing both immunodepression and bacterial infections, which are major causes of morbidity and mortality in this patient population [2–4]. Moreover, acute and chronic peripheral inflammation can stimulate and exacerbate CNS inflammation, potentially leading to the acceleration of preexisting but heretofore latent neurodegenerative disorders [5].

Multiple sclerosis (MS), considered to be a T-cell-mediated autoimmune disease of the CNS, leads to disseminated inflammatory demyelination in the brain and spinal cord [6]. Its pathological hallmark, the demyelinating plaque, is an area of demyelination and gliosis around blood vessels. Acute lesions show perivascular lymphocytes and plasma cells along with infiltrating macrophages that phagocytose myelin membranes. Recent papers report that MS patients run a high risk of bacterial and fungal infections that lead to hospitalization and infection-related mortality [7,8] and some authors are of the opinion that immunotherapy in MS may increase this infection risk [7,8]. Moreover, this autoimmune disease may be potentially triggered by infection, including the Epstein Barr virus, which has been associated with an increased risk of developing MS [9]. In addition, current research suggests that microbial factors might, not only influence the likelihood of developing MS, but also the occurrence of disease exacerbations [10–12].

Polymorphonuclear cells (PMNs) constitute an essential part of the innate immune system and are the first inflammatory cells to migrate towards the site of inflammation [13,14]. Neutrophil granulocytes have also been reported to play an important role in preparing CNS inflammation in the mouse model of MS, in as much as CNS-infiltrating neutrophils secrete proinflammatory molecules and mature bone marrow-derived dendritic cells, which in turn enhance their ability to restimulate myelin-specific T cells [15]. A number of studies report PMN defects in MS patients, although results do vary. PMN functional activities i.e. phagocytosis and the capacity to produce reactive oxygen species (ROS), seem to be particularly altered in MS patients with concomitant bacterial infections [16–18]. However, there is still a paucity of data as to the relationship among alterations of phagocyte functions in MS patients (i.e. cytokine secretion, Toll-like receptor expression pattern, etc.), immunotherapy and microbial factors potentially triggering exacerbations.

The scope of this interdisciplinary preliminary study was to investigate the role PMNs play in patients affected by MS, untreated or treated with immunosuppressant/immunomodulatory drugs, at different disability status, in comparison to a sex- and age-matched control group. To this aim, the *in vitro* ability of patient PMNs to kill *Klebsiella pneumoniae* or *Candida albicans*, to release different cytokines, produce ROS and to regulate apoptosis in the presence of microbial *stimuli*, were evaluated.

## Patients and Methods

### Study design

The study, performed according to the principles of good clinical practice and in line with the Declaration of Helsinki Principles, included peripheral blood analysis with PMN functional determinations. A total of 46 MS patients who presented to the neurology Units of ASL TO3—Rivoli Hospital (Rivoli, Turin, Italy); ASL TO4—Chivasso Civic Hospital (Chivasso, Turin); A.O. Città della Salute e della Scienza di Torino—Molinette Hospital, Turin, over a period of 30 months were enrolled into this cohort study. The control population was made up of 30 healthy subjects (HSs), matched for both age and sex with the MS patients, all negative for bacterial and/or viral infections in progress without any history of inflammatory disorders. A written informed consent was obtained from all MS patients and HSs before the blood was drawn. The study protocol was approved of by the Ethical Committees (Protocol 11246, number 51/2009 and Protocol 68195, number CS/249) and for ethical reasons, the trial was limited to adult individuals.

The diagnostic criteria for MS included clinical and paraclinical laboratory assessments to demonstrate lesion dissemination in both space and time (by magnetic resonance imaging–MRI criteria) and to exclude alternative diagnoses. The neurology units recorded all the clinical data including age at onset of MS, disease course, disease duration, disability according to the Kurtzke Expanded Disability Status Scale (EDSS). Information as to infectious complications was also documented to distinguish patients with recurrent infections from those without. Inclusion criteria were: clinically definite MS, aged 18–69 years, disease duration 0–33 years, EDSS scores <6.0. Males and females of all ethnicities were included.

Any MS patients that had not been treated by steroids for at least 4 weeks prior to enrolment or any immunomodulatory or immunosuppressive agent during the previous 6 months, were considered untreated and, therefore eligible to be included in the study.

### PMN functional activity

#### Blood samples

Blood samples from the MS patients and the HSs were collected under the routine sterile conditions at the three neurology units taking part in the study and where immediately forwarded to the Microbiology laboratories of the Department of Public Health and Paediatrics, University of Torino, for *in vitro* assays.

#### Microorganisms

Clinical strains of *K*. *pneumoniae* and *C*. *albicans*, both pathogens commonly found to be responsible for frequent infections in MS patients, were chosen for the *in vitro* assays. The strains were cultured on differential and selective agar suitable for microbial growth: MacConkey agar for *K*. *pneumoniae* and Sabouraud agar for *C*. *albicans* (Merck, Darmstadt, Germany). Young colonies (18–24 hours) were inoculated into cryovials and maintained at -80°C for extended storage.

#### Phagocyte preparation and culture PMNs

Peripheral venous blood from MS patients and HSs were collected into sterile vacuum tubes containing lithium heparin (15 UI LH/ml blood) and settled at room temperature by gravity for 30 minutes in 2.5% dextran (500,000 molecular weight) in standard saline solution (ratio1:1). The leucocyte-rich plasma supernatant was carefully layered on Ficoll-Paque and centrifuged twice at 1,200g for 15 minutes. Pure PMNs were obtained by lysing residual erythrocytes using hypotonic shock for 30 seconds in sterile distilled water, which were then further centrifuged. Following Bürker chamber counting (Marenfield, Germany), PMN density was adjusted to 10^6^ cells/ml in phosphate buffered saline supplied with 0.1% glucose and 0.1% human albumin. The PMNs were placed into sterile tubes in RPMI 1640, supplemented with 10% fetal calf serum. The viability was assayed by trypan blue exclusion before and after each experiment and was greater than 95%. The time lapse between blood sampling and the beginning of the experiments was <3 hours. The time interval between the harvesting of the PMNs and the start of experiments was < 30 minutes [19].

#### Evaluation of intraPMN antimicrobial activity

The phagocytic intracellular microbial killing activity of both MS and HSs patients was investigated by incubating microorganisms and phagocytes at a ratio of 10:1, for *K*. *pneumoniae* and at ratio of 1:1, for *C*. *albicans*, at +37°C for 30, 60 and 90 minutes. Intracellular killing was assessed as previously described [19,20]. PMN killing values were expressed as the survival index (SI), which was calculated by adding the number of surviving microorganisms at time zero to the number of survivors at time X and dividing by the number of survivors at time zero. According to this formula, if microbial killing was 100% effective, the SI would be 1. Data were expressed as mean ± SEM.

### Pre-treatment assays of HS PMNs with natalizumab

The effects of a highly specific α4-integrin antagonist, natalizumab (NAT—Tysabri, Biogen Idec Limited, Innovation House, Berkshire, UK) on intracellular microbial killing by HS PMNs was investigated by incubating the phagocytes with NAT for 1.5 hours, at the concentrations achieved *in vivo* (25 μg/ml). Microorganisms were added to PMNs after the pre-treatment and incubated at +37°C in a shaking water bath for periods of 30, 60 and 90 minutes. The microbicidal activity of PMNs was determined as aforementioned.

### Quantification of cytokine production

PMNs (obtained from both MS patients and HSs) were stimulated by *K*. *pneumoniae* (ratio 10:1) or *C*. *albicans* (ratio 1:1) for 30, 60, 90 and 180 minutes. PMN culture supernatants were assayed to evaluate proinflammatory cytokine production i.e. TNF-α; IL-1β, IL-8, by ELISA kits purchased from R&D Systems (USA) and used according to the manufacturer’s instructions.

### Oxidative burst assay

The neutrophil ROS production was determined during the respiratory burst, 100 μl of heparin-anticoagulated whole blood samples were incubated in polypropylene tubes with 5 μM PMA (Sigma Aldrich, USA), *K*. *pneumoniae* (10:1), *C*. *albicans* (1:1), or left untreated, at +37°C for 30 minutes. Then dihydrorhodamine 123 (DHR) solution (Sigma Aldrich, USA), at a concentration of 30 μg/ml, was added to the stimulated and resting samples. After 5 minutes at +37°C, a FACS lysing solution (Becton Dickinson, USA), was added to all tubes to lyse erythrocytes and the cells were then left at room temperature for 20 minutes. After centrifugation, the samples were resuspended in 1 ml of 1% paraformaldehyde to stabilize the cells and then analyzed by flow cytometry using a FACSCalibur CellQuest (BD Biosciences, USA). A total of 10,000 events was collected for each sample and neutrophils were identified by their typical forward and sideward scatter characteristics.

### Phenotypic analysis of PMNs apoptosis by flow cytometry

Purified PMNs were collected in polypropylene tubes at a concentration of 1x10^6^/ml and left unstimulated or stimulated with *K*. *pneumoniae* (10:1) for 30 minutes. The cells were then washed and left to rest for 3 hours at +37°C in RPMI 10% FCS, after which they were washed, resuspended in Annexin Buffer and stained with FITC-Annexin V and propidium iodide (PI) (Biolegend, USA), the latter to allow for exclusion of necrotic cells, for flow cytometer analysis of apoptosis.

### Data analysis

To detect differences between MS patients (with different treatment regimens or untreated) and controls (HSs), a statistical analysis was performed by the Graphpad Prism version 6 for Windows (Graphpad Software, San Diego, CA, USA) by one-way analysis of variance (ANOVA), followed by Tukey's multiple comparison test. The unpaired T-Student test was used to detect any differences between NAT pre-treated and untreated HS PMNs. The results were analyzed by descriptive statistics (average values ± SEM); p<0.05 were considered significant.

## Results

### Demographic and clinical information of MS patients

**Table 1**summarizes the demographic and clinical characteristics of relapsing-remitting (RR)-MS patients and the HSs. There were similar demographics for MS patients and HSs. No correlations among clinical data (gender, age at onset of MS, disease duration and disability according to the EDSS) were documented. A correlation between recurrent microbial infections and treatment was evidenced: RR-MS patients treated with immunosuppressive drugs were at higher infection risk than the other MS patients (**Table 1**).

**Table 1:** Demographic and clinical information on the healthy subjects (HSs) and MS patients.

	*HSs* (n = 30)	*MS patients* (n = 46)		
		Treated by		
		Immunosuppressors	Immunomodulators	Untreated
**No. of subjects**	30	13	22	11
**Age (years) (range)**	37.10±2.37 (21–61)	43.00±3.29 (23–57)	47.30±2.07 (22–59)	48.64±3.97 (26–69)
**Female/male**	18/12	7/6	16/6	6/5
**Disease duration (years) (range)**	-	7.39 ±2.43 (0–33)	10.14 ±1.40(0–24)	13.00±3.08(2–31)
**EDSS score**	-	1.5 = 1 case;2.5 = 3 cases;3.5 = 1 case;4 = 1 case;4.5 = 3 case;5 = 3 cases;6 = 1 cases	1.5 = 4 cases;2 = 5 cases;2.5 = 4 cases;3 = 1 case;3.5 = 3 cases:4 = 1 case;4.5 = 3 case;6 = 1 cases	1 = 2 cases;1.5 = 2 cases;2 = 4 cases;2.5 = 2 cases;3.5 = 1 case
**Disease course**	-	RR-MS-patients = 13 cases	RR-MS-patients = 22 cases	RR-patients = 11 cases
**Therapy**	-	natalizumab = 6 cases; cyclophosphamide = 6 cases;fingolimod = 1 case	interferon = 6 cases; glatiramer acetate = 16 cases	-
**Recurrent microbial infections**	-	UTIs = 6 cases;RTIs = 2 cases	-	RTIs = 1 case

Average ± SEM are shown; EDSS: Kurtzke Expanded Disability Status Scale; RR: relapsing-remitting MS; UTIs: urinary tract infections; RTIs: respiratory tract infections

### PMN intracellular killing activity against *K*. *pneumoniae* and *C*. *albicans*

The MS neutrophil count, isolated from the peripheral venous blood, overlapped that of HSs. The PMN viability remained unchanged throughout all experiments.

The microbicidal activity towards intracellular *K*. *pneumoniae* by RR-MS PMNs differed according to the presence/absence of treatment. As reported in **Table 2**, HS PMNs were able to kill ingested klebsiellae within 60 minutes; RR-MS PMNs collected from untreated patients displayed an intracellular killing activity that overlapped that of the controls with no statistically significant differences. On the other hand, the microbicidal activity of RR-MS PMNs from patients who had been given either immunosuppressive or immunomodulatory treatment, was statistically significantly (*p*≤0.001) inhibited within the 60 minutes of incubation.

**Table 2:** PMN intracellular killing activity (%) against *K*. *pneumoniae* in RR-MS patients and HSs.

	*SURVIVAL INDEX (average ± SEM)*				
	Healthy subjects (n = 30)	Untreated RR-MS patients (n = 11)	TreatedRR-MS patients		Statistical analysis
			Immunosuppressive treatment(n = 13)	Immunomodulatory treatment(n = 22)	Tukey's multiple comparison test
	A	B	C	D	
**30’**	1.58±0.02(42%)	1.58±0.04(42%)	1.83±0.04(17%)	1.75±0.04(25%)	p<0.0001 A *vs*. C; p = 0.0001 A *vs*. D;p<0.01 B *vs*. C;p<0.05 B *vs*. D
**60’**	1.75±0.02(25%)	1.76±0.06(24%)	1.98±0.02(2%)	1.96±0.02(4%)	p = 0.0001 A *vs*. C;p<0.001A *vs*. D;p<0.01 B *vs*. C;p = 0.0001 B *vs*. D
**90’**	>2(0%)	>2(0%)	>2(0%)	>2(0%)	n.s.

The percentage of initial microbial population killed by PMNs; statistical significance was p< 0.05

Comparable *in vitro* results were obtained with *C*. *albicans*. PMNs from HSs and from untreated RR-MS patients displaying a similar intracellular killing activity within 90 minutes of observation without statistically significant differences, whereas PMNs from RR-MS patients treated with immunosuppressive or immunomodulatory drugs showed a statistically significantly (p<0.01) lower intracellular killing activity (**Table 3**).

**Table 3:** PMN intracellular killing activity (%) against *C*. *albicans* in RR-MS patients and HSs.

		*SURVIVAL INDEX (average ± SEM)*			
	Healthy subjects (n = 30)	Untreated RR-MS patients (n = 11)	TreatedRR-MS patients		Statistical analysis
			Immunosuppressive treatment(n = 13)	Immunomodulatory treatment(n = 22)	Tukey's multiple comparison test
	A	B	C	D	
**30’**	1.61±0.03(39%)	1.65±0.03(35%)	1.86±0.02(14%)	1.82±0.04(18%)	p<0.0001 A *vs*. C and A *vs*. D;p<0.01 B *vs*. D and B *vs*. C
**60’**	1.60±0.02(40%)	1.68±0.01 (32%)	1.83±0.03(17%)	1.78±0.03(22%)	p<0.0001 A *vs*. C and A *vs*. D;p = 0.0001 B *vs*. C; p<0.05 B *vs*. D
**90’**	1.56±0.03(44%)	1.65±0.02(35%)	1.88±0.02(12%)	1.78±0.04(22%)	p<0.0001 A *vs*. C and A *vs*.D and B *vs*. C;p<0.05 B *vs*. D

The percentage of initial microbial population killed by PMNs; statistical significance was p< 0.05.

### Effect of NAT on HS PMN killing activity

**Fig 1:**
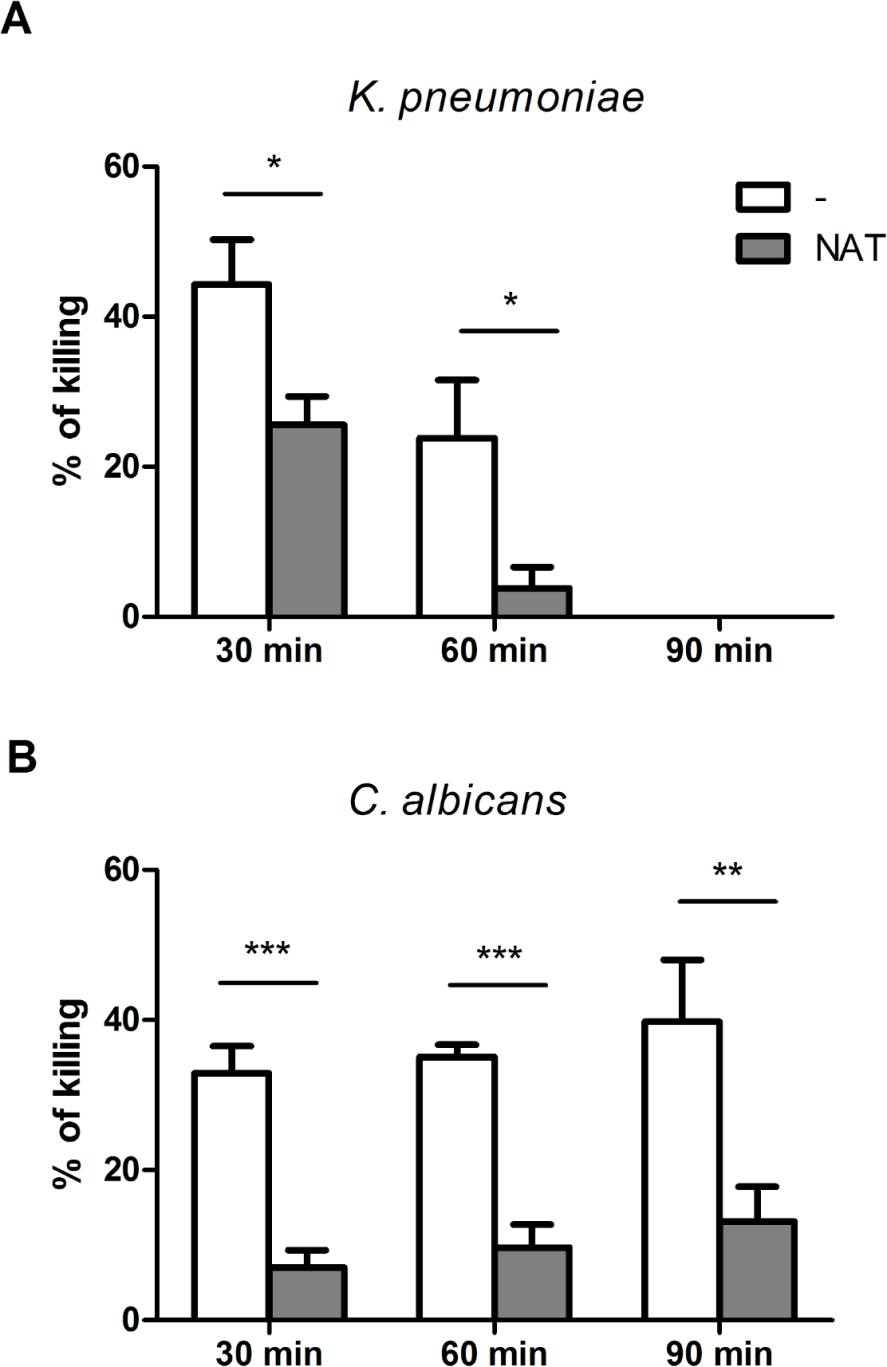
The effect of natalizumab (NAT) on PMN bactericidal activity. PMNs isolated from healthy subjects (n = 6) were pre-treated with NAT for 1.5 hours and then *K*. *pneumoniae* (**A**) or *C*. *albicans* (**B**) killing (displayed as %) was assessed. Data are represented as average ± SEM. * p<0.05; ** p≤0.001; *** p<0.0001.

HS PMNs were pre incubated for 1.5 hours with 25 μg/ml of NAT to investigate the direct effect of the drug on the phagocyte functions. *In vitro* assays with NAT pre-treated PMNs showed a reduction in both bacterial (**Fig 1A**) and fungal (**Fig 1B**) intracellular killing with statistically significantly lower values (p<0.05 and p<0.0001, respectively) than those registered for the non pre-treated controls.

### ROS production and apoptosis of PMNs after microbial exposure

**Fig 2:**
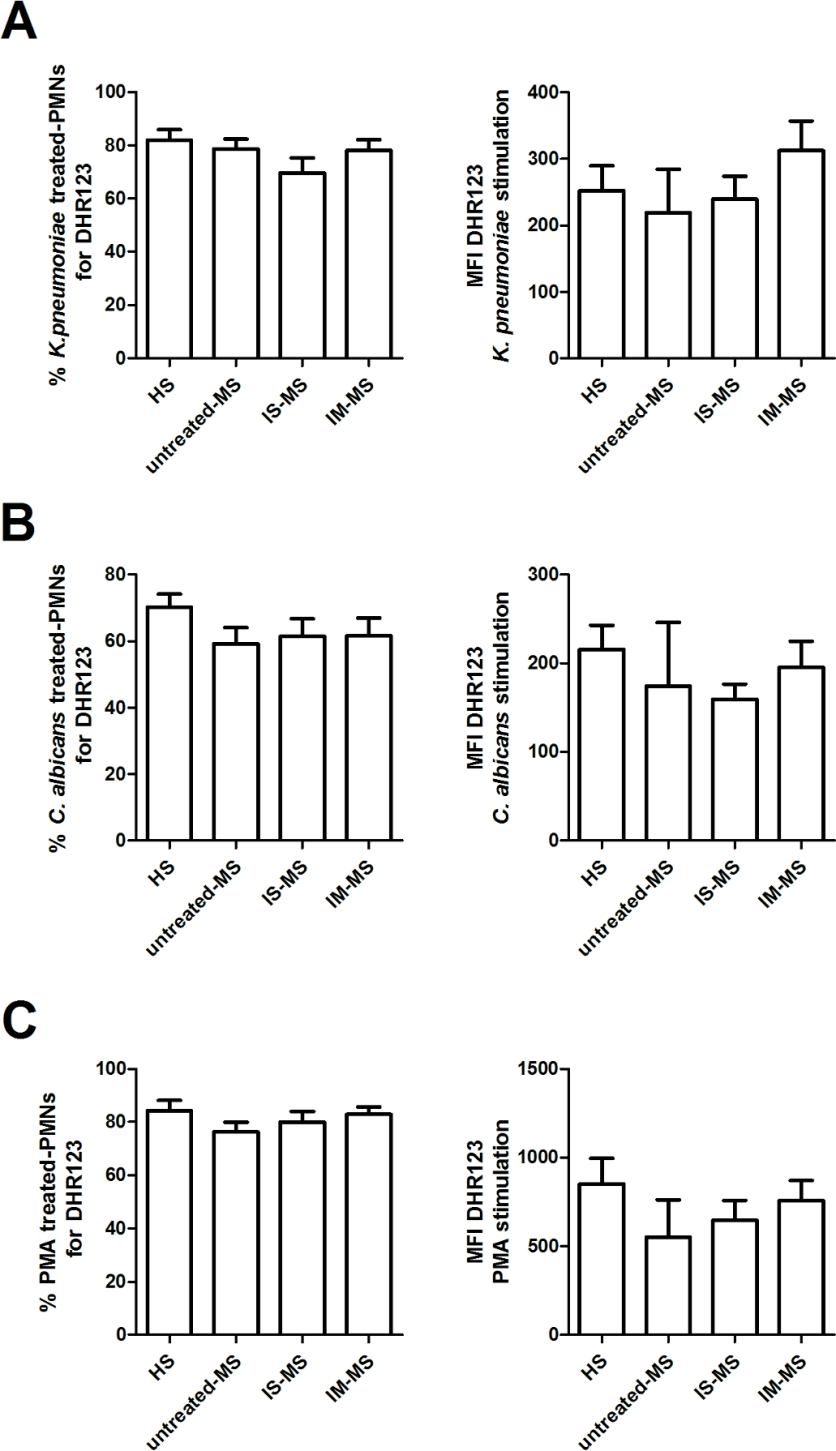
The analysis of induced oxidative burst in MS patients. Whole blood from healthy subjects (HS; n = 12), from untreated-MS patients (n = 7), from patients treated with immunosuppressive drugs (IS-MS; n = 6) or with immunomodulatory drugs (IM-MS; n = 12), was stimulated with *K*. *pneumoniae* (**A**), *C*. *albicans* (**B**) or PMA (**C**) for 30 minutes. Percentage of positive cells for DHR123 (left panels) or average of fluorescence intensity (MFI) of DHR123 (right panels) were determined by flow cytometry. Data are represented as average ± SEM.

The neutrophil intracellular ROS production from untreated or treated RR-MS patients and HSs was examined and compared, to assess whether the generation of ROS is involved in the various intracellular killing activities in MS patients. Superoxide production was measured by flow cytometry in whole blood neutrophils stimulated by *K*. *pneumoniae*, *C*. *albicans*, PMA, or left untreated. The percentage of PMNs that produce ROS in response to the different stimuli was very similar between the groups, irrespective of treatment (**Fig 2**, left panels); these results were also confirmed by the MFI expression (**Fig 2**, right panels).

**Fig 3:**
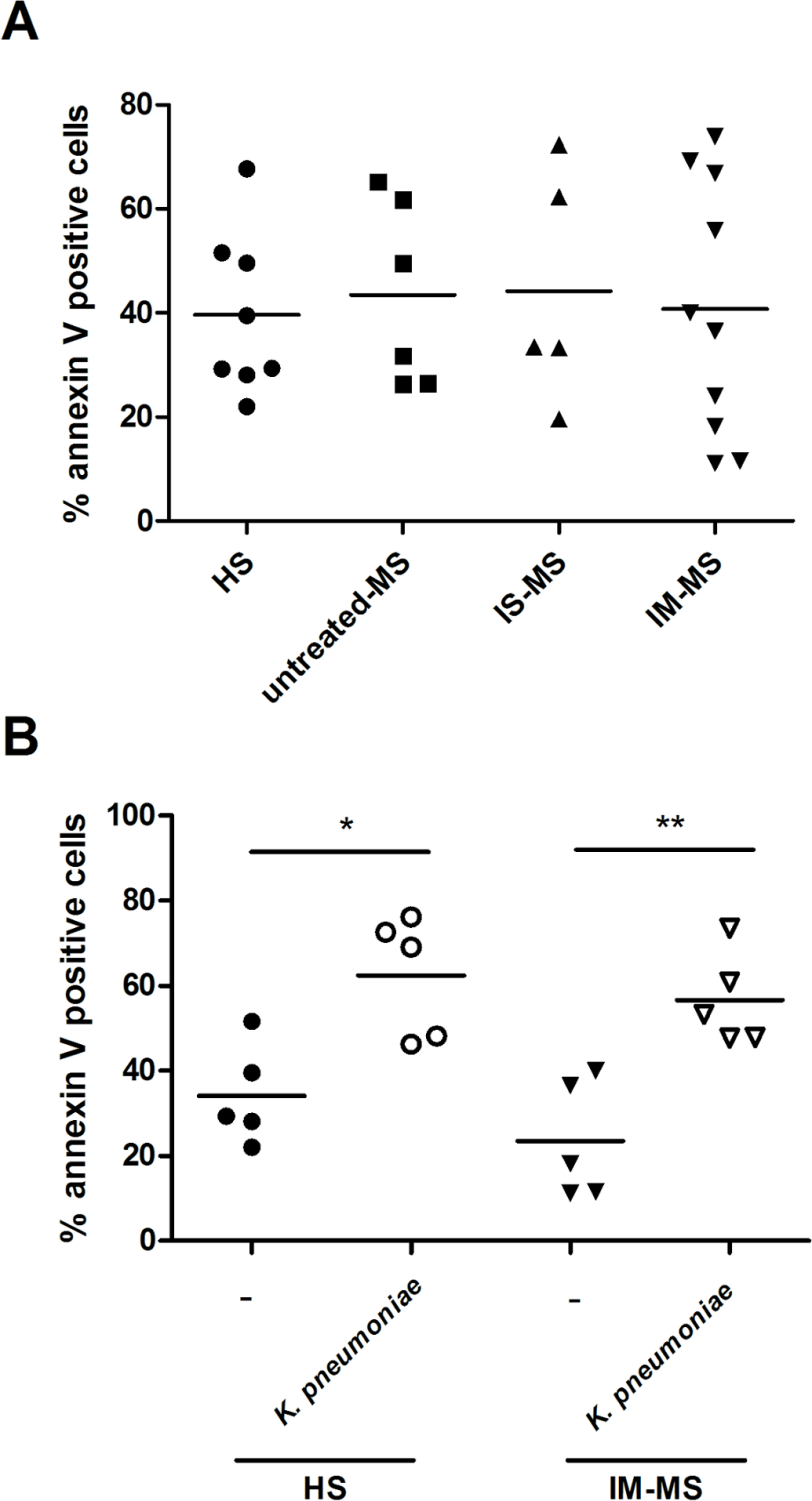
The study of PMN apoptosis in MS patients. (**A**) The percentage of apoptotic purified PMNs (annexin V positive, PI negative) in healthy subjects (HS; n = 8), in untreated-MS patients (n = 6), in patients treated with immunosuppressive drugs (IS-MS; n = 5) or with immunomodulatory drugs (IM-MS; n = 10), after 3 hours of culture. (**B**) Early apoptotic cells were evaluated in HS (n = 5) and IM-MS (n = 5) PMNs after *K*. *pneumoniae* stimulation. Values were determined by flow cytometric analysis and data represented as average ± SEM. *p<0.05; **p<0.001

Neutrophils are characterized by a short lifespan and infection typically accelerates their turnover [21]. Flow cytometry was performed to determine apoptosis in isolated PMNs during basal conditions and after stimulation with *K*. *pneumoniae*. The percentage of annexin V-positive/propidium iodide-negative staining, indicating early apoptosis, demonstrated a similar spontaneous apoptosis in RR-MS PMNs and HS PMNs after 3 hours of incubation (**Fig 3A**). Moreover, a significant decrease in the viability of klebsiella-treated PMNs compared to unstimulated cells was observed both in HSs and RR-MS patients, whether treated or not with immunosuppressive or immunomodulating drugs (**S1 Table** and **Fig 3B**), even if differences without statistical significance were noted in all neutrophils groups.

### PMN cytokine release pattern after bacterial and fungal exposure

**Fig 4:**
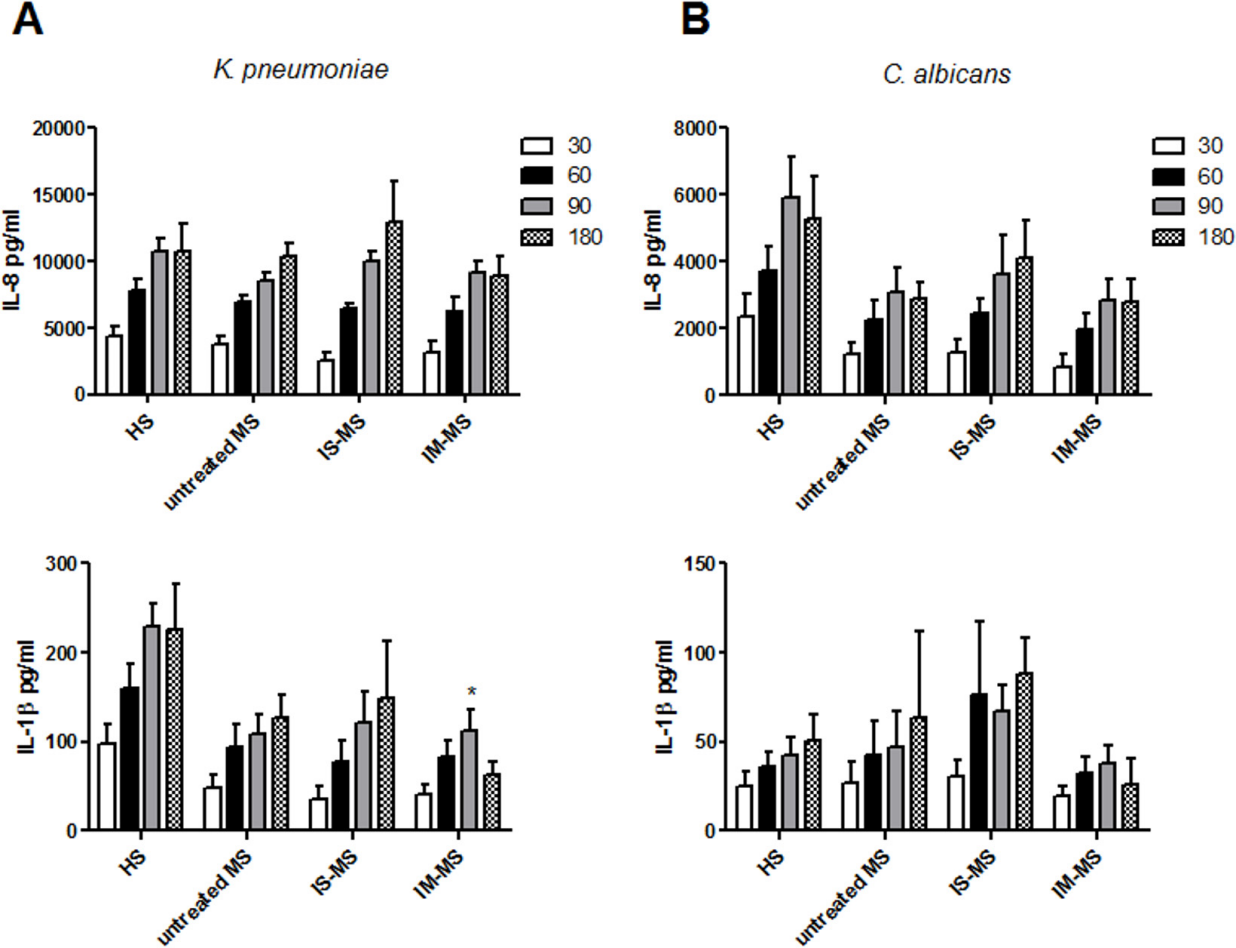
Proinflammatory cytokine release by PMNs. (**A**) PMNs from 16 healthy subjects (HS), from 4 untreated-MS patients, from 6 patients treated with immunosuppressive drugs (IS-MS) or from 13 patients treated with immunomodulatory drugs (IM-MS) were stimulated with *K*. *pneumoniae* for 30, 60, 90 and 180 minutes. (**B**) PMNs from 10 healthy subjects (HS), from 7 untreated-MS patients, from 7 patients treated with immunosuppressive drugs (IS-MS) or from 9 patients treated with immunomodulatory drugs (IM-MS) were stimulated with *C*. *albicans* for 30, 60, 90 and 180 minutes. Supernatants were evaluated for IL-8 (upper panels) and IL-1β release (lower panels). Data are represented as average ± SEM. *p<0.05.

PMNs are normally involved in the initiation of local immune responses by the production of a host of cytokines and chemokines [22]. Therefore, the ability the PMNs of HSs and RR-MS patients had to produce the pro-inflammatory cytokines IL-1β and TNF-α and the chemokine IL-8 following *K*. *pneumoniae* and *C*. *albicans* stimulation was investigated. The results showed a progressive increase in IL-8 and IL-1β production from 30 to 180 minutes in both HSs and RR-MS patients. A lower level of IL-8 production following *C*. *albicans* stimulation was observed in all RR-MS PMNs than in HS PMNs, without statistical significance (**Fig 4**). A slight reduction in the release of IL-1β was detected in RR-MS PMNs after stimulation with *K*. *pneumoniae*; however, this result was statistically significant for only one time point (90 minutes) in those patients treated with immunomodulatory drugs. TNF-α production in PMNs treated with *K*. *pneumoniae* was similar for all groups; no TNF-α was detected after stimulation with *C*. *albicans* (**S1 Table**).

## Discussion

Although MS is not considered a lethal disease, secondary complications, such as infections and respiratory diseases, are common causes of death in MS patients [8]. Literature data suggest that MS could be induced and/or exacerbated by microbial infections. The agents responsible for this are most likely ubiquitous pathogens that are highly prevalent in the general population [23,24]. The infection risk among MS patients has potential implications for clinical management and resources with a heavy socioeconomic impact and is of concern in as much as infections may exacerbate the neurological progression and the development of new symptoms, increasing the risk of acute attacks in RR patients [7,8,25]. Based on current data, microbial infections have been reported to have a strong causal association with death in MS patients, although to date their frequency has not yet been thoroughly investigated and, therefore, remains underestimated [25].

PMNs are key defenders against microbial infection as they eliminate pathogens through various effector mechanisms. These include engulfment and intracellular degradation of microbes through an array of both oxygen-independent and oxygen-dependent killing mechanisms, such as the release of antimicrobial and proteolytic peptides into the phagosomal space, along with the production of reactive oxygen species by the NADPH oxidase complex [14,16]. To date there is a paucity of literature on PMN activity in MS patients and the conclusions reached are heterogeneous. Naess A. et al. were the first to evaluate granulocyte functions, such as motility and activation, in MS patients. These authors reached the conclusion that granulocytes from MS patients function normally [26]. On the contrary, Podikoglou et al., showed that PMNs typical function like adherence, chemotaxis, phagocytosis and bactericidal activity are significantly diminished in MS patients [18]. More recently, Naegele et al. reported that the chronic inflammatory environment in MS probably underlies an inappropriate neutrophil priming, which may lead to an increased neutrophil activation during infection [16].

The data from our study, related to PMN functional activities in patients with RR-MS, a condition that affects the vast majority (85–90%) of MS patients, indicate that PMNs from control HSs and untreated RR-MS patients displayed a similar, or slightly lower, intracellular killing activity within 90 minutes of incubation, with no statistically significant differences against both *K*. *pneumoniae* and *C*. *albicans*. Conversely, PMNs from RR-MS patients treated either by immunosuppressive or immunomodulatory drugs, showed a significantly lower intracellular killing activity against both pathogens. The lower PMN intracellular killing activity detected in treated RR-MS patients suggests that this defect might well play a key role in increasing infection risk in these patients and although it seems to be strongly correlated to the RR-MS treatment, it is independent from their EDSS score status. Following advances in the understanding of the immunological mechanisms that underlie the pathogenesis of MS, a growing arsenal of immunomodulatory agents is available, ranging from non-selective immunosuppression to highly specific immune intervention [27]. Targeted forms of immunosuppression have been referred to as immunomodulatory therapies. As with immunosuppressive regimens, many of these immunomodulatory medications, such as NAT, a new addition to the armamentarium for treating MS, may also have the unintended *consequence* of favouring the development of microbial infections [23,28]: recurrent microbial urinary and respiratory tract infections were reported in our MS patients, mainly in those treated with immunosuppressive therapy. In this context, so as to assess if the therapy was the causal factor for the decline in PMN killing activity observed in treated RR-MS patients, preliminary *in vitro* assays with HS PMNs pre-treated with NAT were carried out. Drug pre-treated HS PMNs showed a reduction in PMN bacterial/fungal intracellular killing, with significantly lower values than those registered for untreated controls. Our *in vitro* pre-treatment results are in contrast with those of Fleming et al., who reported that anti-α4β1 integrin antibody induces receptor internalization and does not alter rat neutrophil phagocytic activity, or oxidative activity when exposure times are brief [29]. However these results may be attributable to the different methodologies used and also to the fact that Fleming’s experiments were performed on animal models.

Therefore, so as to investigate into the mechanisms underlying the killing activity of PMNs in RR-MS patients, we evaluated the oxidative burst. No statistically significant difference was observed in the production of intracellular ROS in RR-MS PMNs, whatever the treatment regimen, compared to HSs in response to PMA or microbial stimuli. An increase in the oxidative burst in MS patients was previously observed by Naegle et al. [16] and Ferretti et al. [17]. This discrepancy between their results and ours may well be due to differences in the number, ages and MS status of the patients. Moreover, our study evaluated the patients according to treatment regimen.

Neutrophil programmed cell death is essential for the resolution of inflammation and abnormal neutrophil apoptosis is associated with a decrease in antimicrobial defences [30]. However, no alterations in the apoptosis of the PMNs of all groups were observed under both stimulated and unstimulated conditions.

Phagocyte populations cooperate to eliminate bacterial invaders and maintain inflammation through the generation of pro-inflammatory cytokines, such as TNF-α, IL-1β, IL-8, until such time as the pathogens have been eliminated [31]. Our results showed that, compared to HS PMNs, there was a slight reduction in IL-1β and IL-8 production after *Klebsiella* and *Candida* infection respectively, in all groups of MS patients.

Microbe killing is a critical physiological function of PMNs, involving various mechanisms and although ROS and cytokine production do not seem to have an impact on the differences observed in the defective killing capacity of treated-MS PMNs, other mechanisms might be involved.

We acknowledge that this study does have some limitations. Firstly, only MS patients with RR disease course, the most common type of MS, were recruited. Secondly, as it was difficult to find untreated MS, there were very few in our cohort. Lastly, the direct effect of the therapy on PMN functional activities was evaluated by only one immunosuppressive drug, excluding the other available immunomodulatory and immunosuppressive therapies for MS. Further experiments with other immunomodulatory drugs, such as interferon β-1b on ongoing in an effort to support the data obtained in this pilot study.

In spite of these limitations, we may deduce that the PMN impairment detected in the treated RR-MS patients may be an additional cause for the incidence of microbial infections in MS patients. However, our data must be considered tentative pending results from additional studies that will hopefully provide a more through understanding of the complex interplay among MS, innate immune system and therapy regimens: including *i)* a wider cohort of treated and untreated patients affected by the most common types of MS; *ii)* other MS phagocytes such as monocytes and dendritic cells, besides PMNs; *iii)* the evaluation of phagocytosis and superficial receptor expression; *iv) in vitro* phagocyte pre-treatment in healthy subjects with the most common immunosuppressant or immunomodulating drugs currently used for MS therapy.

The validation of these results could help in identifying a subset of patients at high risk of infection who could benefit from a closer follow-up and/or antibiotic prophylaxis.

## Supporting Information

S1 Table RR-MS patient and HS minimal data set of the study. Table reporting demographic information, PMN intracellular killing activity, PMN oxidative burst, PMN apoptosis and PMN proinflammatory cytokine release.
(CSV)
